# First Case of Actinomycetoma in France Due to a Novel *Nocardia* Species, *Nocardia boironii* sp. nov.

**DOI:** 10.1128/mSphere.00309-16

**Published:** 2016-11-23

**Authors:** Jacques M. Gilquin, Brigitte Riviere, Valme Jurado, Bernard Audouy, Jean-Baptiste Kouatche, Emmanuelle Bergeron, Delphine Mouniée, Thierry Molina, Philippe Faure, Cesáreo Saiz-Jimenez, Verónica Rodríguez-Nava

**Affiliations:** a Service de Médecine Interne, Centre Hospitalier de Castres-Mazamet, Castres, France; b Laboratoire de Microbiologie, Centre Hospitalier de Castres-Mazamet, Castres, France; c Instituto de Recursos Naturales y Agrobiologia, IRNAS-CSIC, Seville, Spain; d Service d’Imagerie Médicale, Centre Hospitalier de Castres-Mazamet, Castres, France; e Research Group on Bacterial Opportunistic Pathogens and Environment, UMR 5557, Ecologie Microbienne, French Observatory of Nocardiosis, Université de Lyon, Université de Lyon, CNRS, VetAgro Sup, Lyon, France; f Service d’Anatomie et de Cytologie Pathologiques, Hôpital Necker Enfants Malades, Paris, France; g Laboratoire d'Anatomie et de Cytologie Pathologiques, Castres, France; University of Kentucky

**Keywords:** MLSA, *Nocardia*, antibiotic resistance, genotypic identification, mycetoma, phenotypic identification, taxonomy

## Abstract

Bacterial mycetoma is a neglected disease mainly observed in tropical area countries and typically associated with rural conditions, making its presence in developed countries of temperate climate areas rare. However, we report the first case of an autochthonous mycetoma case in continental France that originated from a new *Nocardia* species. A Gram-positive filamentous bacterium (OFN 14.177^T^) was isolated from a pus sample from the mycetoma of a male French patient 92 years old suffering from chronic lymphocytic leukemia. The isolate was analyzed by a polyphasic taxonomic approach by coupling morphological, biochemical, physiological, and chemotaxonomic aspects to genomic and phylogenetic analyses. Multilocus sequence analysis (MLSA) using four housekeeping genes (16S rRNA gene, *secA1*, *hsp65*, and *sod*) combined with phylogenetic analysis revealed that the strain OFN 14.177^T^ is phylogenetically closer not only to *Nocardia altamirensis* but also to all other species comprising the *Nocardia brasiliensis* clade (i.e., *N. brasiliensis*, *N. altamirensis*, *N. vulneris*, *N. iowensis*, and *N. tenerifensis*), some of which present cutaneous tropism. The G+C content of isolate OFN 14.177^T^ was 68.2 mol%. DNA-DNA hybridization analyses demonstrated 38.25% relative reassociation with *N. altamirensis*. The strain OFN 14.177^T^ is different from the closest species at genetic and phenotypical levels, and the data obtained indicate that it should be recognized as a new species, for which the name of *Nocardia boironii* sp. nov. is proposed. The type strain is OFN 14.177^T^ (= EML 1451 = DSM 101696).

**IMPORTANCE** Bacterial mycetoma is an endemic infection in areas with tropical and subtropical climates. Thus, its presence in temperate climate areas remains rare. We report here the first case of autochthonous actinomycetoma in continental France originating from a *Nocardia* species other than *N. brasiliensis*, namely, *Nocardia boironii*. Considering the history of the patient, the infection source of strain OFN 14.177^T^ may be from frequent contact with the soil over many years because of his gardening activities. The discovery of a French autochthonous *Nocardia* species responsible for actinomycetoma reveals the importance of considering the possibility of having autochthonous infections of this type in nontropical countries, not only imported cases from tropical countries. However, further studies are needed to elucidate the real incidence of this new species.

## Abstract (precis)

Bacterial mycetoma is an endemic infection in areas with tropical and subtropical climates. Thus, its presence in temperate climate areas remains rare. We report here the first case of autochthonous actinomycetoma in continental France originating from a *Nocardia* species other than *N. brasiliensis*, namely, *Nocardia boironii*. Considering the history of the patient, the infection source of strain OFN 14.177^T^ may be from frequent contact with the soil over many years because of his gardening activities. The discovery of a French autochthonous *Nocardia* species responsible for actinomycetoma reveals the importance of considering the possibility of having autochthonous infections of this type in nontropical countries, not only imported cases from tropical countries. However, further studies are needed to elucidate the real incidence of this new species.

## INTRODUCTION

*Nocardia* bacteria are widely present in the environment, and several species are responsible for different types of infections in humans and animals (1–3). *Nocardia* bacteria are opportunistic pathogens (4) responsible for systemic nocardiosis (skin, lung, abdomen, brain, etc.) and primitive cutaneous infections. The cutaneous form of infection is typically observed in immunocompetent patients following traumatic injury. There are different types of cutaneous infection: superficial, deep, and lymphocutaneous. The diagnosis of mycetoma in underdeveloped countries where modern laboratory methods are lacking can be difficult and may be given several years after the primary infection. The primary infection occurs mainly on the feet and arms (5).

In this work, we analyze a strain obtained from a mycetoma sample that exhibited atypical morphological and genetic characteristics. The taxonomic position of this strain was researched by modern taxonomic methods. The data obtained showed that this strain should be recognized as a new species, for which the name *Nocardia boironii* sp. nov. is proposed.

### Strain.

The strain (OFN 14.177^T^) was isolated from a pus sample from a French patient suffering from mycetoma at the CHIC Castres-Mazamet microbiology laboratory in Castres, France.

### Case report.

The patient, a 92-year-old former gardener, went to the emergency department with shortness of breath, cyanosis, and signs of acute heart failure. He had a background history of asymptomatic chronic lymphocytic leukemia (CLL) (Rai stage 0), coronary stent implantation for myocardial infarction, and benign prostatic hypertrophia. His current medications consisted of clopidogrel, bisoprolol, furosemide, and ramipril. The family of the patient reported a progressively reduced general condition, significant weight loss, and the presence of skin lesions on the forearm with purulent discharges that had occurred for an extended period of time.

**FIG 1:**
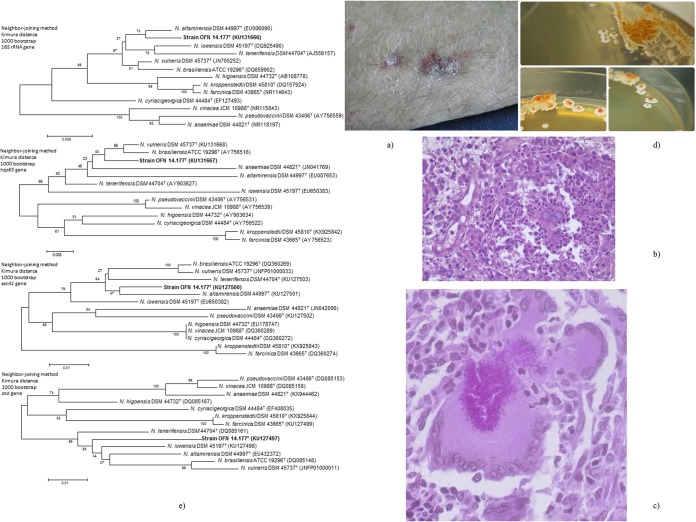
(a) Mycetoma aspect of the patient in the right forearm. Two purplish nodular lesions of 1 to 2 cm over a small indolent subcutaneous tumefaction of 2 by 3 cm and an indurated scar on the right elbow. (b) Histologic findings revealed a suppurative granuloma surrounding grains stained with periodic acid-Schiff (PAS) stain; granulomas were composed of neutrophils surrounded by a mixed inflammatory infiltrate comprising lymphocytes, macrophages, and numerous multinucleated giant cells. (c) At a higher magnification, histologic findings showed short hyphae that could sometimes be observed within the giant cells (PAS stain). (d) The morphology of colonies of the isolate OFN 14.177^T^ on Bennett agar after 10 days at 28°C. Regarding the OFN 14.177^T^ strain, we obtained yellow/orange and dome-shaped colonies 1 to 3 mm in diameter, exhibiting a rough and dry aspect and a slight aerial mycelium at the colony surface after 7 days of culture on Bennett agar at 28°C. (e) Phylogenetic trees based on the 16S rRNA, *hsp65*, *secA1*, and* sod* gene sequences of our OFN 14.177^T^ strain and the closest *Nocardia* type strains. These trees are based on the analysis of a 1,325-nt fragment of the 16S rRNA gene, a 401-nt fragment of the *hsp65* gene, a 551-nt fragment of the *secA1* gene, and a 444-nt fragment of the *sod* gene. Evolutionary trees were obtained from the distance matrix by the neighbor-joining method (16). The bootstrap values were calculated by random resampling of the sequences (*n* = 1,000) to obtain the most representative phylogenetic tree. The bars show 0.005 or 0.01 nucleotide substitutions per position.

Upon physical examination, his temperature was 38.5°C, and he had a pulse rate of 95/min, blood pressure of 120/80 mm Hg, and oxygen saturation of 85% while breathing ambient air. There were crepitations at both pulmonary bases, edema of the legs, and hepatojugular reflux. Details about the skin examination are presented in Fig. 1a. There was no lymphadenopathy. The remainder of the examination was normal.

Laboratory results revealed a hemoglobin level of 13.8 g/dl, platelet count of 293 × 10^9^/liter, white cell count of 37.4 × 10^9^/liter (27% lymphocytes, 67% granulocytes), C-reactive protein level of 203 mg/liter, creatinine level of 268 µmol/liter (clearance modification of diet in renal disease [MDRD] of 20 ml/min), and normal hemostasis and liver parameters.

Amoxicillin-clavulanic acid (AMC) (given intravenously), O_2_ nasal administration, and furosemide treatment were initiated before the patient was transferred to the Medicine Department with a diagnosis of bronchopulmonary infection and heart failure.

A computed tomography scan of the thorax revealed alveolar condensation in the right inferior lobe associated with bronchiolar bilateral nodules in the upper lobes and a small bilateral pleural effusion without mediastinal lymphadenopathy.

The skin lesions associated with intermittent purulent discharge began on the right elbow more than 15 years ago. The lesions on the right forearm were observed 3 years ago.

A nodule biopsy sample was taken by a dermatologist 1 year ago. The histologic findings are shown in Fig. 1b and c. Surprisingly, no microbiological investigations were performed. Thus, we informed our microbiological laboratory of a strong suspicion of actinomycetoma before performing an ultrasound-guided fine needle aspiration of the subcutaneous tumefaction.

### First isolation from clinical specimen.

The bacteria in the pus sample were cultured on Columbia blood agar, Columbia ANC blood agar, chocolate Polyvitex, and Drigalski media at 37°C in an aerobic atmosphere with 5% CO_2_. *Nocardia*-like small yellow colonies formed 7 days later on Columbia agar and chocolate Polyvitex plates.

### Treatment and effect on patient.

Progressive clinical improvement regarding arm lesions was observed after the initiation of a treatment with AMC. We then decided to continue the same antibiotic for 6 months. The first microbiological investigations were in favor of a *Nocardia* infection, and cerebral magnetic resonance imaging revealed no cerebral localization. After 2 months, a complete resolution of the pulmonary lesions was observed on a computed tomography (CT) scan. The cutaneous nodules improved much more slowly and evolved toward purplish scars. No relapse was observed 1 year after the end of the treatment. No pulmonary investigation was made before the initiation of AMC given the severe hypoxemia; however, nocardial pneumopathy appears improbable in this patient presenting with a long history of mycetoma. The infection was cutaneous and subcutaneous without extension to muscle and bone despite CLL-associated immunodeficiency.

### Taxonomy.

*Nocardia boironii* (boi.ron’ii. NL gen. masc. n. boironii of Boiron, was named in honor of Patrick Boiron, a French microbiologist, for his enormous contributions to the taxonomy and epidemiology of *Nocardia*).

**FIG 2:**
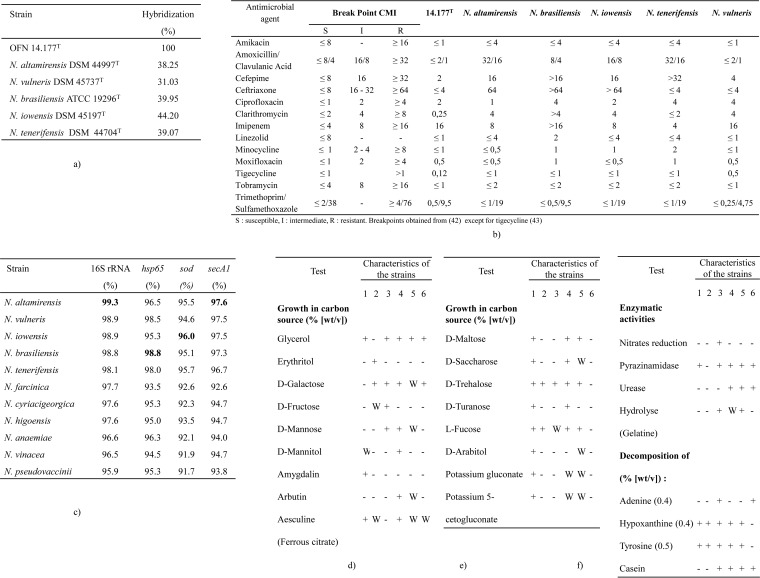
(a) DNA-DNA hybridizations of strain OFN 14.177^T^ with the species having the highest similarity percentages in the 16S rRNA, *hsp65*, *sod*, and* secA1* genes. (b) Susceptibility of strain OFN 14.177^T^ and type strains of genetically closest species against antibiotics. The MIC (CMI) break points were obtained from reference 42 except for those for tigecycline (43). -, no data. (c) Similarity percentages of the sequences of the rRNA 16S, *hsp65*, *sod*, and *secA1* genes between strain OFN 14.177^T^ and the phylogenetically closest species. The highest similarity values for each gene are indicated in boldface type. (d to f) Physiological characteristics of the OFN 14.177^T^ strain and *Nocardia* reference type strains (strain 1, *N. boironii* sp. nov. OFN 14.177^T^; strain 2, *N. altamirensis* DSM 44997^T^; strain 3, *N. vulneris* DSM 45737^T^; strain 4, *N. brasiliensis* ATCC 19296^T^; strain 5, *N. iowensis* DSM 45197^T^; strain 6, *N. tenerifensis* DSM 44704^T^). Growth in carbon source and decomposition are shown as percentages (wt/vol). Reactions: −, negative; +, positive; W, weak.

An aerobic, Gram-positive bacterium, positive for catalase and presenting filamentous thin branched bacilli that easily fragment into bacillus- or coccus-shaped elements. The color of the mycelium is yellow-orange. The aerial mycelium is white with a nonhomogeneous distribution. The colonies measure 1 to 3 mm in diameter on Bennett agar. Growth occurs at 28°C and 37°C, but not at 45°C. Major fatty acids include palmitic acid (C_16:0_) (46.69%), tuberculostearic acid (C_10-Me-18:0_) (22.86%), and oleic acid (C_18:1w9c_) (12.29%). The mycolic acids are 46 to 60 carbon atoms in length (the main mycolic acids have a chain length of 52 and 54 carbon atoms). The DNA G+C content of the type strain is 68.2 mol%. Physiological and biochemical characteristics of strain OFN 14.177^T^ are presented in Fig. 2d, e, and f.

The type strain OFN 14.177^T^ (= EML 1451^T^ = DSM 101696^T^) was isolated from a pus sample from the mycetoma of a French patient (Castres, France).

## RESULTS AND DISCUSSION

Bacterial mycetoma, which is often observed in tropical areas (6), exhibits a particular case of cutaneous and subcutaneous damage. With slow and painless progression in approximately 60% of the cases, it can be provoked by different actinobacteria species (*Nocardia*, *Actinomadura*, and *Streptomyces*) (7). However, the agents responsible for human mycetoma vary according to the geographic region. In the case of *Nocardia*, the responsible species are mainly *N. brasiliensis*, followed by the species belonging to the former *N. asteroides* complex (*N. cyriacigeorgica, N. abscessus, N. transvalensis-N. wallacei* complex) (8) and more recently, *N. mexicana* (9), *N. harenae* (10), and *N. takedensis* (11), or *N. africana* in animals (12).

Mycetoma remains localized, but it can spread through tissues. With time, the infection reaches the muscle and bone, in which destructive osteomyelitis can occur. These clinical manifestations are observed in Mexico, South America, and Africa in immunocompetent patients who have a rural way of life with difficult access to health centers (13, 14). Walking barefoot in the fields or forests may favor repeated bacterial inoculation.

The aspect of OFN 14.177^T^ strain culture in Bennett agar is presented in Fig. 1d. After Gram staining the culture, we observed thin Gram-positive branched filaments compatible with the *Nocardia* genus. Modified Ziehl-Neelsen staining revealed partially acid-alcohol-resistant filaments for OFN 14.177^T^ strain, which is characteristic of the *Nocardia* genus.

### Chemotaxonomic and genetic characterization.

Analysis of the OFN 14.177^T^ strain cell wall composition revealed mycolic acids with a chain length of 46 to 60 carbon atoms. This strain also presented as main menaquinone MK-8 (H_4_, cycl). The main fatty acids present in the strain were palmitic acid (C_16:0_) (46.69%), tuberculostearic acid (C_10-Me-18:0_) (22.86%), and oleic acid (C_18:1w9c_) (12.29%). These chemotaxonomic properties are consistent with those described for the *Nocardia* genus by Goodfellow et al. (15) and Minnikin et al. (16), which support the assignment of this strain to the *Nocardia* genus.

The G+C content of the strain OFN 14.177^T^ DNA was 68.2 mol%. Phylogenetic analysis revealed that the nucleotide sequence of the gene coding for the 16S rRNA of strain OFN 14.177^T^ exhibited a sequence similarity of 99.3% with *N. altamirensis* DSM 44997^T^ as the closest species, followed by *N. vulneris* DSM 45737^T^ and *N. iowensis* DSM 45197^T^ with 98.9% sequence similarity. Phylogenetic analysis led us to classify our strain inside a taxonomic clade that is represented by *N. brasiliensis* (i.e., *N. brasiliensis*, *N. altamirensis*, *N. iowensis*, *N. tenerifensis*, and *N. vulneris*) (Fig. 1e). Many of the species of this clade present a cutaneous tropism (14, 17, 18).

Genetic analysis to make a correct identification at the species level using the 16S rRNA gene revealed that the closest species *N. altamirensis* DSM 44997^T^ did not exhibit sufficient similarity with the OFN 14.177^T^ strain, according to CLSI MM18 (19) identification guidelines (99.6%). Moreover, OFN 14.177^T^ strain seemed close to *N. brasiliensis* according to its morphological characteristics. For these reasons, we decided to proceed to multilocus sequence analysis (MLSA) identification using other genes, such as *hsp65*, *sod*, and *secA1*.

Genetic analysis with these genes revealed that the most related species to the OFN 14.177^T^ strain were different according to the gene used, and their similarity values were even lower than that obtained using 16S rRNA gene, as noted in Fig. 2c. However, in every case, the OFN 14.177^T^ strain remained inside the same *N. brasiliensis* clade.

Historically, it was considered that DNA-DNA hybridization (DDH) is necessary to confirm the presence of a new species when 16S rRNA similarity is greater than 97% between two strains (20). On the other hand, Goris et al. (21) compared the DDH technique with obtained values of genome sequence-derived parameters, such as the average nucleotide identity (ANI) of common genes. ANI is a robust measurement of genomic relatedness that represents a mean value of the similarity of homologous genomic regions of two genomes (22). Goris et al. stated that 70% of genetic relatedness using DDH corresponded to 95% ANI. Later, Kim et al. (22) enhanced the work of Goris et al. by analyzing up to 22 phyla of prokaryotes for which the complete genome was available and found that 95% ANI similarity corresponded to a threshold of 98.65% of 16S rRNA gene similarity.

In our study, we obtained 16S rRNA gene similarity values greater than 98.65% between our strain and the phylogenetically closest species, but low similarity values with other housekeeping genes prompted us to hypothesize that our strain may correspond to a new species; thus, DDH or ANI studies with the phylogenetically closest species were necessary. In our case, a DDH was performed because the complete genome of the strain was not available.

The results of DDH with the phylogenetically closest species yielded hybridization values less than 70% as shown in Fig. 2a. These results confirmed that strain OFN 14.177^T^ represents a novel genospecies that is clearly differentiated from all the phylogenetically closest species.

Our study shows that even with 16S rRNA gene similarity values greater than the threshold of 98.65% suggested by Kim et al. (22), the bacterium under study may belong to a new species, and the MLSA technique may be useful to confirm this suspicion.

### Physiological characterization.

Growth of strain OFN 14.177^T^ occurs at 28°C and 37°C, but not 45°C. Our strain exhibits physiological characteristics that are different from those of the phylogenetically closest *Nocardia* type strains. For example, strain OFN 14.177^T^ can use amygdalin, arabitol, potassium gluconate, and potassium 5-cetogluconate as carbon sources, but not d-galactose, as shown in Fig. 2d, e, and f.

### Antimicrobial susceptibility test.

The results of the antibiogram performed using the broth microdilution method on our strain and those of species from the *N. brasiliensis* clade are presented in Fig. 2b.

Drugs normally used in probabilistic antibiotherapy for nocardiosis (trimethoprim-sulfamethoxazole, amikacin) are effective against *N. boironii*. Moreover, amoxicillin-clavulanic acid, cefepime, ceftriaxone, clarithromycin, linezolid, minocycline, moxifloxacin, tigecycline, and tobramycin could also be a treatment choice for this new species. On the other hand, this species exhibited intermediate resistance against ciprofloxacin and resistance against imipenem.

This study allowed us to confirm that the strain OFN 14.177^T^ belongs to a new species of the *Nocardia* genus according to the polyphasic approach, which encompassed genetic, chemotaxonomic, phenotypic, and drug susceptibility analyses. The name *Nocardia boironii* sp. nov. is proposed.

## MATERIALS AND METHODS

### Growth and morphology studies.

The OFN 14.177^T^ strain was cultivated on Bennett’s agar at 37°C and 28°C for 1 week to observe the morphological characteristics and aerial hyphal production. For better characterization of the strain according to its morphological features, we performed Gram staining and modified Ziehl-Neelsen staining (23).

### Chemotaxonomic analyses.

The fatty acid and mycolic acid composition was determined by the Deutsche Sammlung von Mikroorganismen und Zellkulturen using the standard Microbial Identification System (MIDI) (24). Isoprenoid quinones were extracted from freeze-dried biomass of strain OFN 14.177^T^ using the small-scale procedure of Minnikin et al. (16, 25), separated by high-pressure liquid chromatography and analyzed by the method of Kroppenstedt (26, 27).

### G+C content of DNA.

The method of Mesbah and Whitman (28) has been used for the GC calculation of deoxyguanosine (dG) and thymidine (dT) ratio.

### Multilocus sequence analysis (MLSA) and phylogeny.

Phylogenetic analysis of four different genes (16S rRNA gene, *hsp65*, *sod*, and *secA1*) was performed to characterize the OFN 14.177^T^ strain. An almost complete sequence of the 16S rRNA gene (1,325-nucleotide [nt] fragment) was determined using SQ1 (5′-AGAGTTGATCMTGGCTCAG-3′) and SQ6 (5′-CGGTGTGTACAAGGCCC-3′) primers, as described by Rodriguez-Nava et al. (9). The partial sequences of three housekeeping genes were also determined for the gene coding for the heat shock protein (*hsp65*), the gene coding for the superoxide dismutase (*sod*), and the gene coding for the preprotein translocase subunit ATPase (*secA1*), according to the guidelines of Rodriguez-Nava et al. (9, 29, 30) and Conville et al. (31). To perform the phylogenetic analysis, we obtained the sequences of the type strains of *Nocardia* species genetically closest to strain OFN 14.177^T^ from the GenBank database with the exception of *N. vulneris*, *N. altamirensis*, *N. iowensis*, *N. pseudovaccinii*, and *N. anaemiae*, which we determined.

The multiple-sequence alignment software Clustal X (32) and the MEGA6 software (33) allowed us to build phylogenetic trees by three different methods: maximum likelihood (34), maximum parsimony (35), and neighbor joining (36). Branch robustness was calculated by a random resampling according to the bootstrap method (1,000 replications).

### DNA-DNA hybridization (DDH).

The degree of DNA-DNA relatedness between strain OFN 14.177^T^ and all the species belonging to the *N. brasiliensis* complex (i.e., *N. altamirensis*, *N. vulneris*, *N. iowensis*, *N. tenerifensis*, and *N. brasiliensis*) was determined using the digoxigenin labeling/antibody detection system described by Ziemke et al. (37) and Urdiain et al. (38). DNA was labeled and detected colorimetrically using *p*-nitrophenyl phosphate as the substrate. Chromosomal DNA was extracted by the method of Marmur (39).

### Physiological and biochemical characteristics.

The methods of Boiron et al. (23), Goodfellow et al. (15, 40), and Goodfellow and Lechevalier (41) were used to determine the decomposition of adenine, casein, hypoxanthine, testosterone, tyrosine, uric acid, and xanthine, assimilation of substrates, growth in different carbon sources (glycerol, erythritol, etc.), and production of enzymatic activities. Strain OFN 14.177^T^ and the strains of species belonging to the *N. brasiliensis* clade (*N. brasiliensis*, *N. altamirensis*, *N. iowensis*, *N. tenerifensis*, and *N. vulneris*) were tested. Strain OFN 14.177^T^, *N. brasiliensis* ATCC 19296^T^, and* N. vulneris* DSM 45737^T^ were incubated at 37°C, and *N. altamirensis* DSM 44997^T^*, N. tenerifensis* DSM 44704^T^, and* N. iowensis* DSM 45197^T^ were incubated at 28°C.

### Antimicrobial susceptibility test (broth microdilution).

Susceptibility testing was performed using the CLSI (M24-A42)-recommended broth microdilution MIC method (42). The OFN 14.177^T^ strain and all the strains phylogenetically close to this strain were assessed by this method. This test was performed with the recommended primary and secondary antimicrobials (Fig. 2e). MICs were also interpreted according to the CLSI guidelines (42).

### Accession number(s).

The nucleotide sequences determined for *N. boironii* sp. nov. OFN 14.177^T^ were submitted to the GenBank database under accession numbers KU131666 (16S rRNA gene), KU131667 (*hsp65* gene), KU127497 (*sod* gene), and KU127500 (*secA1* gene).
